# Maternal Infection and Preterm Birth: From Molecular Basis to Clinical Implications

**DOI:** 10.3390/children10050907

**Published:** 2023-05-22

**Authors:** George Daskalakis, Alexandros Psarris, Antonios Koutras, Zacharias Fasoulakis, Ioannis Prokopakis, Antonia Varthaliti, Christina Karasmani, Thomas Ntounis, Ekaterini Domali, Marianna Theodora, Panos Antsaklis, Kalliopi I. Pappa, Angeliki Papapanagiotou

**Affiliations:** 1 First Department of Obstetrics and Gynecology, ‘Alexandra’ Hospital, Medical School, National and Kapodistrian University of Athens, 157 72 Athens, Greece; gdaskalakis@yahoo.com (G.D.); psarris.alexandros@gmail.com (A.P.); antoniskoy@yahoo.gr (A.K.); hzaxos@gmail.com (Z.F.); ioannisprokopakis@gmail.com (I.P.); antonia.varthaliti@hotmail.com (A.V.); ckarasmani@gmail.com (C.K.); thomasntounis@hotmail.com (T.N.); kdomali@yahoo.fr (E.D.); martheodr@gmail.com (M.T.); panosant@gmail.com (P.A.); kalliopi.pappa20@gmail.com (K.I.P.); 2 Department of Biological Chemistry, Medical School, National and Kapodistrian University of Athens, 157 72 Athens, Greece

**Keywords:** preterm birth, prematurity, infection, inflammation

## Abstract

As the leading cause of neonatal morbidity and mortality, preterm birth is recognized as a major public health concern around the world. The purpose of this review is to analyze the connection between infections and premature birth. Spontaneous preterm birth is commonly associated with intrauterine infection/inflammation. The overproduction of prostaglandins caused by the inflammation associated with an infection could lead to uterine contractions, contributing to preterm delivery. Many pathogens, particularly *Chlamydia trachomatis*, *Neisseria gonorrhoeae*, *Trichomonas vaginalis*, *Gardnerella vaginalis*, *Ureaplasma urealyticum*, *Mycoplasma hominis*, *Actinomyces*, *Candida* spp., and *Streptococcus* spp. have been related with premature delivery, chorioamnionitis, and sepsis of the neonate. Further research regarding the prevention of preterm delivery is required in order to develop effective preventive methods with the aim of reducing neonatal morbidity.

## 1. Introduction

Preterm birth (PTB), defined as birth prior to 37 weeks’ gestation, is the main cause of neonatal death, as 27% of neonatal mortality is related to complications of PTB [1]. According to the World Health Organization, the global annual burden of PTB is estimated to be 15 million [2]. The incidence of PTB has been calculated at 12.7 % in the United States of America, while other developed countries such as Sweden, Japan, Australia, and New Zealand have PTB rates between 4.4 and 8.2% [3,4,5]. Regional variations are similarly clear in the European Union, where preterm birth rates range between 5 and 10% [6]. According to the most recent data from the Hellenic Statistical Authority (ELSTAT), the number of preterm births in Greece increased to 12,831 (11.18%) in 2010 [7]. At the same time, data suggest that prematurity is higher in the non-Hispanic Black population (16.75% compared with 10.49% for the non-Hispanic White population) [8]. Prematurity is a major cause of infant mortality, while sequelae due to preterm birth are usual in the neonatal period and may remain into adulthood [9].

Preeclampsia or intrauterine growth restriction are common reasons for iatrogenic PTB [10,11], while multiple causes such as immunological disorders, infection/inflammation, uterine overdistension, and vascular disease are considered responsible for spontaneous preterm births [12]. In addition, periodontal disease, uteroplacental ischemia and hemorrhage, shortened cervical length, polyhydramnios, multiple gestation, poor maternal nutritional status, and racial disparity are other risk factors for PTB [12,13,14,15,16,17,18].

A significant percentage of PTB, ranging between 25 and 40%, has been attributed to infections, both overt and subclinical [12]. Sometimes, it is unclear whether the infections are a cause of PTB or part of the processes resulting in PTB. However, both microbiological and biochemical data suggest that an important percentage of preterm deliveries can be attributed to both infections and the inflammation caused by infections. Firstly, the higher levels of inflammatory cytokines found in the amniotic fluid of patients with preterm labor are a clear indicator [19]. Secondly, the microbial colonization of women with PTB has been shown to differ between women not in labor and women laboring at term [19]. According to in vitro studies, prostaglandin E2 levels are raised after amnion cells are exposed to bacterial products [20]. Furthermore, the administration of microbes or microbial products to pregnant animals has resulted in preterm labor [21,22]. Concurrently, subclinical uterine infections have also been related to PTB [23]. In other studies, the presence of an intra-amniotic infection or intrauterine inflammation during the second trimester has been shown to increase the risk for PTB [24]. Lastly, premature parturition has been associated with extrauterine maternal infections such as periodontal disease, pneumonia, and pyelonephritis [25,26,27].

In many cases, spontaneous preterm labor is a syndrome attributable to multifactorial inflammatory mechanisms. These inflammatory processes may lead to preterm premature rupture of membranes (PPROM). Positive amniotic fluid cultures and histological chorioamnionitis are more common in PPROM patients than in normal controls [28,29].

The purpose of this article is to review what is currently known about maternal infections and their impact on pregnancy outcomes.

## 2. Inflammation in Labor and Preterm Labor

Numerous studies on preterm birth have shown that term and preterm labor share the same underlying process, with the only distinction being the gestational age at which labor begins. It is thought that both conditions share a common pathway. The pathological processes that lead to preterm labor involve the activation of one or more components of this common pathway, such as the increased production of prostaglandins and proteases in the genital tract; the functional withdrawal of progesterone due to a decreased expression of progesterone receptor (PR) isoforms in the cervix, decidua, and myometrium; and changes in hormone concentrations such as corticotropin-releasing factor (CRF) and cortisol [30,31,32].

Preterm birth has also been linked to maternal and fetal stress. Corticotropin-releasing hormone (CRH) seems to be the mediator of preterm births caused by stress. During pregnancy, the hypothalamus and placental, chorionic, amniotic, and decidual cells all produce the peptide hormone CRH, which is composed of 41 amino acids. Both maternal and fetal stress can elevate CRH levels, resulting in increased cortisol levels in both the mother and the fetus. By upregulating the expression of cyclooxygenase-2 (COX-2) and inhibiting prostaglandin dehydrogenase (PGDH), elevated cortisol levels may increase the release of prostaglandins (PGs) by the fetal membranes. In addition, prostaglandins promote cervical changes and PPROM by increasing the expression of matrix metalloproteinases (MMPs) in the genital tract, increasing the gap junction between uterine cells, promoting the formation of myometrial oxytocin receptors, and suppressing myometrial PR expression. PGs also increase the cervical expression of interleukin-8 (IL-8), causing neutrophils to release additional MMPs and elastases [33,34,35,36]. MMPs degrade and decompose collagen, with their activity increasing in the fetal membranes during PPROM and labor. 

Inflammation can be considered a regulative mechanism by which the tissues respond to injurious stimuli in order to control and repair possible damage. Whether arising from periodontitis, pneumonia, cholecystitis, pyelonephritis, pancreatitis, sepsis, or genital tract inflammatory states such as bacterial vaginosis, deciduitis, chorioamnionitis, or intra-amniotic infections, it has been associated with preterm birth (PTB) [37,38]. This is because inflammation induces an exaggerated immune response that increases the production of inflammatory cytokines, elastases, and MMPs (matrix metalloproteinases) and induces the functional withdrawal of progesterone, a vital hormone for maintaining pregnancy. In pregnancy, the inflammatory response can be deemed a theoretical model in which the infected cavity evacuates any products that put at risk the health of the mother, so that the reproductive capacity is preserved for the future.

Genetic predispositions to inflammation, including specific gene polymorphisms such as the TNF-a gene T2 allele, which increases the risk of preterm premature rupture of membranes (PPROM) in African American women, or polymorphisms in TLR-4 (a significant endotoxin-signaling receptor), have also been linked to PTB. Polymorphisms in drug-metabolizing genes such CYP1A1, HincII RFLP, and GSTT1 have been linked to PTB in Chinese women who were exposed to benzene and in American women subjected to cigarette smoke. African American fetuses at risk for PPROM carry mutations in the MMP-1 and MMP-9 genes [39,40,41,42].

Bacteria are found in the fetal circulation in 30% of incidents of intra-amniotic infection, leading to a systemic inflammatory response in the fetus. Due to the immaturity of multiple organ systems, these fetuses are at risk for long-term complications, inversely correlated with the gestation age, such as cerebral palsy and respiratory and gastrointestinal complications, underlining that it is not only the immaturity that is responsible for the complications of infants born preterm but also the inflammatory process [43,44].

Microorganisms, including those of the lower genital tract, have been isolated from amniotic fluid, suggesting that the most common route of infection is an ascending one. [45]. Bacteria linked to periodontal disease have been detected in amniotic fluid, suggesting the possibility of hematogenous dispersion with transplacental passage [46]. Preterm birth has also been linked to infections, which has been linked to invasive medical operations [47].

Human parturition is an inflammatory process. An alteration from an inactive to a pro-inflammatory environment signals the initiation of labor, which is characterized by three steps: uterine contractility, cervical ripening, and membrane activation and rupture. It is believed that the beginning of term labor is the result of processes like progesterone withdrawal, oxytocin secretion, decidual triggering, and activation of the fetal immunological response [12].

Several hypotheses have been developed on the association between spontaneous preterm labor and infection. Most likely pathways for infection-induced PTB include decidual stimulation and the fetal immunological response, both of which are triggered by the innate immune system′s reaction to infection. [17]. Microorganisms and their products that reach the amniotic cavity are sensed by transmembrane pattern recognition receptors (PRR), such as acute phase receptors and toll-like receptors (TLRs), which are bound to patterns of molecular structures on the surface of the microorganisms. There are 11 distinct TLRs found in humans, and they all have a role in controlling inflammation. [48]. TLR-4 has a role in the immune response to lipopolysaccharides (LPS) and the byproducts of Gram-positive bacteria, mycoplasmas, and yeast [49]. It has been established that PTB is linked to increased expression of TLR-2 and TLR-4 in the chorioamniotic membranes, both of which have been identified in the amniotic epithelium [50]. TLR ligation induces the synthesis of cytokines (IL-1b, IL-6, TNF-a, granulo-cyte colony-stimulating factor, or tumor necrosis factor-a) and chemokines (IL-8, MCP-1) by activating nuclear factor kappa B and other kinases inside the cell [48,51]. These substances promote both the stimulation of neutrophils and the production of prostaglandins, trigger the uterine contractions, and induce the metalloproteinase-induced membrane damage in PPROM and PTB [14]. Experimental evidence suggests that TLRs play a vital role in the genesis of spontaneous preterm labor (SPTL), and defective signaling through TLRs weakens defenses against PTB caused by bacteria [52].

Moreover, a fetal response takes place as the infection promotes the release of corticotropin-releasing hormone and subsequently the release of fetal corticotropin and fetal cortisol from both the placenta and the fetal hypothalamus, resulting in prostaglandin production [18]. In inflammation- or infection-induced preterm delivery, both pro- and anti-inflammatory cytokines play critical roles [16]. When an infection causes premature labor, IL-1 is the first cytokine to be involved. By increasing the synthesis and activity of COX-2, which increases myometrial contractions, it is released by stimulated monocytes and macrophages [52]. 

IL-6, IL-16, IL-18, colony-stimulating factors, IL-8, and monocyte chemotactic protein-1 are all pro-inflammatory cytokines thought to play a role in the development of PTB. In addition, Gram-positive bacterial infections trigger death of tropho-blast cells by activating Toll-like receptors 1 and 2. Apoptosis is induced in neutrophils and T cells after they have been stimulated to undergo chemotaxis and activation by IL-6 and IL-8 secreted by trophoblast cells. This could be a fundamental process underlying SPTL. IL-10, an anti-inflammatory cytokine, is found to be reduced in the placenta in term patients, suggesting that its downregulation during labor induces the inflammatory process that is necessary for parturition. Inhibiting IL-12 and IFN-g production, as well as the stimulation of T cells, monocytes, and macrophages, IL-10 exerts powerful immunosuppressive activity, making it essential for pregnancy maintenance [52,53]. Moreover, IL-4 downregulation promotes spontaneous abortion and preterm birth [54]. Nuclear factor-B (NF-B) is regulated by and activates pro-inflammatory cytokines. It has a crucial role in the processes associated with labor. Abnormal activation of NF-B contributes to the initiation of PTL, as it is important for the production of prostaglandins and MMP expression, and subsequently, for the stimulation of uterine contractions and cervical ripening [55].

Choriodecidual space infections, amnion infections, chorion infections, placenta infections, amniotic fluid infections, umbilical cord infections, and fetal infections are all possible during pregnancy [14].

Chorioamnionitis is an inflammation of the amniotic sac and placenta caused by an infection of the fetus. It often takes years for symptoms of many infections to manifest. Histological chorioamnionitis is frequently linked to preterm births before 30 weeks of gestation in about 50% of cases [56]. Histopathology is the gold standard for confirming intrauterine infections, however clinical, biochemical, and microbiological parameters have also been used. However, not enough research has been done to fully understand how the immune system reacts to various infections during chorioamnionitis.

PTB risk factors also include vaginal bleeding, placental abruption, decidual hemorrhage, and other vasculopathies. Decidua, the uterine lining during pregnancy, is an abundant source of tissue factor, and decidual hemorrhage results in an increased production of thrombin, which binds to its proteinase-activated receptor, resulting in an increased expression of MMP-1 and mRNA in the decidual cells. Decidual neutrophils, which are frequently found in regions of thrombin-induced fibrin deposition and thrombin/PAR-1, promote the expression of IL-8, mRNA, and inflammatory proteins. Neutrophils are also an abundant source of elastases and MMP-9, which can cause premature membrane rupture (PROM) and cervical effacement [57,58,59].

Mechanical extension of the uterus, such as mechanical dilation of the cervix, has also been linked to an increased risk of PTB because it induces the expression of prostaglandins and MMP-1. Other factors, such as polyhydramnios (excessive amniotic fluid) and multifetal gestation, can also enhance the expression of the inflammatory enzyme COX-2. The expression of oxytocin receptor, COX-2, IL-8, and connexin is induced by myometrial stretch, which is the elongation of the uterine muscle layer [60].

Various modalities have been employed to prevent preterm birth, and these modalities are frequently incorporated into the biochemical processes implicated in preterm birth. Antibiotics have been used, for instance, to treat maternal infections, thereby reducing the inflammatory response and the risk of preterm birth. Tita et al. discovered, through a systematic review and meta-analysis, that antibiotics given to expectant women with bacterial vaginosis, asymptomatic bacteriuria, or group B *Streptococcus* colonization were associated with a significant reduction in preterm birth [15].

Progesterone supplementation has also been shown to prevent preterm birth, particularly in women with a history of spontaneous preterm birth or a short cervix [61]. Progesterone has anti-inflammatory properties and can aid in the maintenance of uterine quiescence, thereby preventing cervical maturation and the uterine contractions that occur prematurely [62]. In a meta-analysis, Romero et al. revealed that women who already had a record of spontaneous preterm birth benefited greatly from progesterone supplementation [63].

Cervical cerclage has been extensively utilized as a preventive modality in women with cervical insufficiency to reinforce the cervical integrity and prevent premature cervical dilation [64]. Berghella et al. showed in a meta-analysis and comprehensive review that women with cervical insufficiency who receive cervical cerclage have a much lower risk of having a premature baby [65]. Other preventive modalities, including cervical pessaries and lifestyle interventions, have also been investigated as potential interventions to prevent preterm birth [66].

In addition, recent developments in molecular biology and genetics have illuminated potential preterm birth prevention targets. The inhibition of specific inflammatory pathways, such as the NF-B pathway, has shown promise in preclinical investigations as a potential strategy to reduce preterm birth associated with inflammation [67]. Similarly, targeting specific hormonal pathways, such as the progesterone receptor signaling pathway, may provide new therapeutic approaches for the prevention of preterm birth [68].

**Figure 1:**
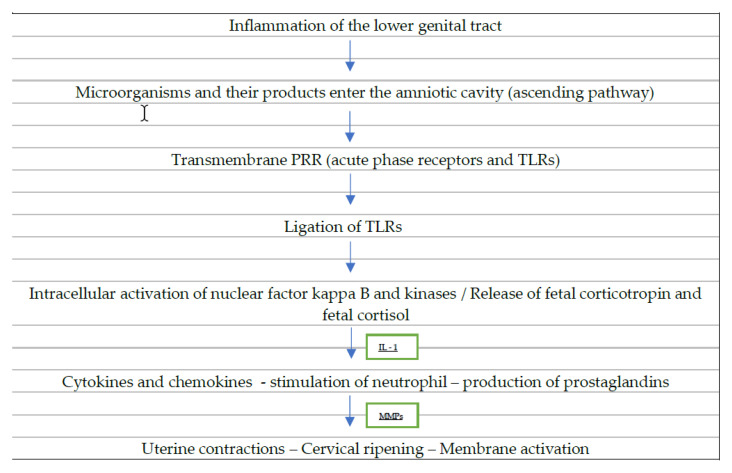
Inflammation leading to preterm labor. Possible mechanisms through which inflammation can lead to preterm labor (PRR: pattern recognition receptors, TLRs: toll-like receptors, IL-1: interleukin 1, MMPs: matrix metalloproteinases).

Recent NGS-based research, such as Fettweis et al.’s 2019 *Nature Medicine* article, has shed light on the links between aberrant vaginal microbiotas and preterm births. These studies have demonstrated that an imbalance in the vaginal microbiome, which includes a decrease in *Lactobacillus species* and an increase in diverse anaerobic bacteria, increases the risk of preterm birth, chorioamnionitis, and neonatal sepsis. *Gardnerella*, *Prevotella*, and *Ureaplasma* are associated with adverse outcomes. The timing of microbial colonization in pregnancy is important because an aberrant vaginal microbiome in early pregnancy is linked to a higher risk of unfavorable outcomes than one later in gestation. These NGS-based findings strongly imply that targeted therapies to alter the vaginal microbiome could avert unfavorable neonatal outcomes. However, additional investigation is needed to identify the best measures and to comprehend the complicated connections between the microbiome of the vagina and perinatal outcomes [69] (Figure 1).

## 3. Specific Infectious Organisms

Many different types of bacteria have been linked to preterm birth, chorioamnionitis, and early-onset neonatal sepsis. These include *Gardnerella vaginalis*, *Ureaplasma urealyticum*, *Mycoplasma hominis*, *Chlamydia trachomatis*, *Trichomonas vaginalis*, *Neisseria gonorrhoeae*, *Actinomyces*, *Candida* spp. However, the role of the individual pathogens as the culpable organisms is less clear.

### 3.1. Bacterial Vaginosis (BV)

Bacterial vaginosis is the prevailing lower genital tract disorder among women of reproductive age [70]. This disorder is not caused by a single pathogen, such as in a classical infection, but is the consequence of a modification in the vaginal flora, where physiological lactobacilli are replaced by an overgrowth of mixed flora with increased numbers of the anaerobic bacteria that normally appear in the vagina in smaller amounts, including *Gardnerella vaginalis*, *Bacteroides* spp., *Mycoplasma hominis,* and *Mobiluncus* spp. [71,72].

In normal vaginal flora, the H_2_O_2_ that is produced by *lactobacilli* prevents the overgrowth of anaerobes, acting against the proliferation of other microorganisms by maintaining an acidic vaginal pH. In the presence of BV, there is a shift in the anaerobe-to-aerobe ratio as the vaginal pH increases due to a decreased amount of the produced H_2_O_2_. In BV we found the same kind of bacteria as in the normal flora, but the difference is in the quantity of the present microorganisms [73]. BV is often asymptomatic, although sometimes results in a gray vaginal discharge with a characteristic “fish odor” [69]. Three out of four of the Amsel criteria (vaginal pH > 4.7, presence of discharge, amine odor, and presence of clue cells) or the Nugent score (a Gram-stain grading system) must be met for a classical diagnosis of BV to be made [74,75].

Abortion, PPROM, chorioamnionitis, amniotic fluid infection, and preterm birth are among conditions that have been linked to bacterial vaginosis [76,77]. Fifteen percent to forty-two percent of pregnant women have BV, and it raises the possibility of spontaneous PTB and PPROM by a factor of two to four [28].

Women with or without symptoms are at higher risk of having a premature baby [78]. An increased risk of spontaneous preterm birth has been linked to the presence of certain organisms in BV, including *Ureaplasma urealyticum* and *Mycoplasma hominis* [79]. New hypotheses suggest that BV and premature birth may be influenced by both genes and the environment [69].

However, significant ongoing research is aimed at elucidating the pathophysiologic processes between BV and premature birth. We still don′t know why some women′s BV clears up on its own and others don′t [80]. BV is not often an inflammatory disorder, although many preterm birthing mothers have aberrant vaginal microbiota and an inflammatory process. Women with BV often do not have leukocytes in their vaginal discharge. Pro-inflammatory cytokines like IL-1 are also present, and elevated levels of these cytokines have a role in the local generation of prostaglandin, which has been linked to premature birth [81].

The risk of preterm birth in women may be increased by several factors that act separately or in combination. Firstly, the bacteria of BV ascend to the upper genital tract, causing chorioamnionitis, premature rupture of membranes, and subsequent preterm birth [81]. Moreover, bacteria associated with BV produce proteolytic enzymes that change the permeability of the mucosal epithelium, enabling other, more pathogenic bacteria to cause ascending infections [56]. In addition, in the presence of BV microorganisms, the genetically mediated local innate immunity, producing cytokines, chemokines, and growth factors, has the ability to modulate the response within the vagina and cervix [69,81]. Finally, the degree of difference in the risk of preterm birth may be related either to immune hypo-responses that allow easier ascending infections or to immune hyper-responses in which more inflammatory processes are developed, causing preterm birth [82].

Klebanoff et al.’s 2023 systematic review and individual participant data meta-analysis examined whether BV antibiotics reduce preterm birth risk. The meta-analysis comprised 11 randomized controlled studies with nearly 7000 women. In high-risk populations, the antibiotic treatment of BV resulted in a significant reduction in preterm birth risk. Preterm birth and baseline vaginal pH above 4.5 also were shown to increase the effect. The researchers also warn of antibiotic resistance and the necessity for more research on the best antibiotic regimen and its long-term effects; however, they support the use of antibiotics to treat BV to reduce preterm birth and emphasize the need for more investigation and a thorough examination of the antibiotic treatments’ risks and benefits [83].

### 3.2. Staphylococcus aureus

*Staphylococcus aureus (S. aureus)* is a commensal bacterium and a leading source of invasive infections in humans; it is a well-adapted human pathogen. Nearly 25% of the human population has become persistently colonized, and 75% is intermittently or never colonized [84,85,86,87]. Chorioamnionitis and preterm premature rupture of membranes (PPROM), both of which can lead to preterm birth and neonatal illness, have been linked to S. aureus in recent years. Eleje et al., in a prospective cross-sectional study, found that in women with PPROM, *Streptococcus* species, *S. aureus* and *E. coli* were significantly increased in comparison with women without PPROM [88]. In addition, chorioamnionitis associated with methicillin-resistant *S. aureus* has also been reported [89].

On the surfaces of soft tissues, a biofilm is formed by *S. aureus,* contributing to its resistance to antimicrobials and host immunity [90,91]. *S. aureus* takes advantage of the α-hemolysin pro-inflammatory activity to cause the degeneration of vaginal tissue and improve biofilm generation [92]. Gestational membranes, through receptors that recognize pathogens, contribute to an active immune control [93]. *S. aureus* infection of the gestational membranes produces a biofilm formation that promotes the release of pro-inflammatory cytokines (IL-1β, IL-2, IL-6, GM-CSF, TNF-α, and IFN-γ) [94]. The produced cytokine cascade results in membrane weakening, leading to preterm premature rupture of membranes and preterm birth [14].

### 3.3. Genital Mycoplasmas

Pregnancy is a common time for the isolation of genital mycoplasmas (GMs), which include *Ureaplasma urealyticum* and *Mycoplasma hominis*. Their presence in the amniotic cavity is associated with adverse pregnancy outcomes such as preterm labor, postpartum endometritis, PPROM, neonatal systematic inflammatory response, pneumonia, cerebral palsy, and necrotizing enterocolitis [14,95]. They are the organisms most commonly isolated from the placental membranes and amniotic fluid in both histological and clinical chorioamnionitis [84].

*Mycoplasmas* are closely connected to the epithelium, so they are basically restricted to the mucosal surfaces. The immunogenicity of these attachment molecules permits their adherence to numerous types of cells such as neutrophils, erythrocytes, and epithelial cells, triggering the inflammatory response. Moreover, they cause a direct activation of the complement complex C-1 [96]. In addition, a local cytotoxic outcome is caused by the secretory substances of the *mycoplasmas*, ammonia from the *mycoplasmas*, and urea from the *ureaplasmas*, aggravating the inflammatory response [97]. The immune system is believed to intercede for this response. It is known that *M. genitalium*, using lipopeptides expressed on the cell membrane, stimulates toll-like receptors on epithelial cells, which in turn activate the nuclear factor KB [71]. In a related approach, lipopeptides activate the trophoblasts of full-term placentas, producing cyclooxygenase-2 and prostaglandin E2. In the presence of genital mycoplasmas, pro-inflammatory cytokines, such as IL-1b, IL-6, IL-8, and tumor necrosis factor-a (TNF-a), are increased, promoting the inflammatory response [98].

The frequency of genital *mycoplasmas* depends on socioeconomic status and geographic area, while the detection rate of *U. urealyticum* ranges between 20% and 80% and that of *M. hominis* is estimated to be less than 30% in the data [99,100,101,102,103]. Studies have demonstrated that GMs can fill the amniotic cavity and persist for months, triggering a severe inflammatory response, while being thought of as commensal organisms in the lower genital canal with minimal virulence for infection. In 47% and 30% of verified instances of chorioamnionitis, respectively [100], *U. urealyticum* and *M. hominis* were isolated from placental membranes. The detection of U. parvum in the placental tissue was significantly correlated with acute chorioamnionitis in the women presenting in extreme preterm labor, according to a case control study involving 57 women who delivered before 37 weeks of gestation and who either had (42) or did not have (25) inflammation of the chorioamniotic membranes [97]. In the Alabama Preterm Birth study, the presence of *U. urealyticum* and/or *M. hominis* were higher in cord blood cultures among women with spontaneous PTB compared with those with indicated PTB (34.7% vs. 3.2%; *p* = 0.0001) [84]. In addition, a prospective cohort research analyzed the microbiota profiles of 70 sam-ples from 36 women with PPROM between 24 weeks and 33 weeks and 6 days of pregnancy. Women who tested positive for *Mycoplasma* and/or *Ureaplasma* using polymerase chain reaction (PCR) in this study had babies born at a younger age and weighed less than those born to mothers who tested negative for these pathogens [104]. In addition, Kataoka et al. found that vaginal colonization with *U. parvum*, but not *U. urealyticum*, is associated with late abortion or early preterm birth [105].

Although serological testing is unable to distinguish between preceding and current disease, molecular detection methods are superior to culture-based methods for detection, allowing an increased specificity and discrimination of species and subtypes [106]. The pathogenic role of *Ureaplasma* spp. remains controversial, as the colonization rates of these organisms are usually also very high in normal pregnancies, and *Ureaplasma* infection within placentae is not always associated with inflammation and adverse pregnancy outcomes. Possible explanations include the timing and duration of colonization, differences in virulence between species/strains, interactions with other microorganisms and inflammatory modulators, and lastly, suppression or aggravation via maternal immune responses [107].

Jonduo et al. published a meta-analysis on the connections between commensal vaginal *mycoplasmas*, such as *Mycoplasma hominis*, *Ureaplasma urealyticum*, and *Ureaplasma parvum*, and unfavorable pregnancy outcomes. Most intra-amniotic infections involve these bacteria. Genital *mycoplasmas* were linked to preterm birth, low birthweight, and fetal inflammatory response syndrome. *Ureaplasma urealyticum* and *U. parvum* were particularly related to preterm birth. The meta-analysis suggests targeting genital mycoplasmas to decrease poor pregnancy outcomes; however, the most effective therapies and the complicated relationships between these bacteria and unfavorable pregnancy outcomes need additional research. This meta-analysis also reveals the relevance of commensal *genital mycoplasmas* in unfavorable pregnancy outcomes and emphasizes the need for further investigation [108].

#### Sexually Transmitted Infections

PTB has been linked to several STDs that affect the lower genitalia, such as *Chlamydia trachomatis*, *Trichomonas vaginalis*, and *Neisseria gonorrhoeae*.

### 3.4. Chlamydia trachomatis

*Chlamydia trachomatis* is considered the most commonly isolated sexually transmitted organism. The rate of infection with *C. trachomatis* in pregnancy ranges from 2 to 26% but the prevalence varies within each population [109,110]. Untreated maternal cervical chlamydial infection has been linked to an increased risk of preterm delivery, premature rup-ture of membranes, and perinatal mortality, however this has been disputed by a number of research [111,112], conflicting data exist regarding the association between chlamydia and adverse pregnancy outcome, particularly PTB [113]. A retrospective case control study reported that genitourinary *C. trachomatis* infection in the second trimester increased two- to three-fold the risk of spontaneous PTB, while no association was found between a third-trimester infection and the risk of spontaneous PTB [114]. Olson-Chen et al., in a meta-analysis, provided evidence that chlamydia in pregnancy is associated with a small increase in the odds of multiple adverse pregnancy outcomes [115].

### 3.5. Trichomonas vaginalis

*T. vaginalis* is most common in women of childbearing age; it is believed that up to 25 million pregnant women worldwide are infected with the bacterium [116]. Usually, the protozoan *T. vaginalis* causes an asymptomatic infection, but sometimes symptomatic urethritis, vaginitis, or vulvitis can also occur [117]. Research has associated *T. vaginalis* with premature rupture of membranes, preterm delivery, and low birthweight [118]. In a systematic review and meta-analysis of 11 research, Silver et al. found that *T. vaginalis* during pregnancy was significantly linked with an elevated risk of preterm birth (RR, 1.42; 95% CI, 1.15ߝ1.75; 9 studies; n = 81,101; I = 62.7%). Small-for-gestational-age infants (RR, 1.51; 95% CI, 1.32ߝ1.73; 2 trials; n = 14,843; I = 0.0%) and PPROM were also observed to be significantly higher [119]. Although *T. vaginalis* infection during pregnancy has been linked to preterm delivery, Klebanoff et al. evaluated the effectiveness of a 2 g dosage of metronidazole to that of a placebo, administered 48 hours apart. Management of pregnant women with asymptomatic trichomoniasis did not prevent premature delivery [120,121], the authors stated. The PTB rate was substantially higher in the antibiotic group (19.0% vs. 10.7%).

### 3.6. Neisseria gonorrhoeae

*Neisseria gonorrhoeae*, a sexually transmitted Gram-negative intracellular diplococcal organism, is often considered a risk factor for PTB, but this association has not been examined widely. Only a few studies have reported a connection between *N. gonorrhoeae* and PTB. A population-based cohort study showed that maternal gonorrhea is associated with small-for-gestational-age infants [122]. Moreover, Donders et al. reported that *N. gonorrhoeae* during pregnancy increased the risk of developing PROM compared with not having *N. gonorrhoeae* by six times [123]. However, in another unmatched case control study among women who were in the third trimester of pregnancy, no association was found between *N. gonorrhoeae* and PROM [118]. Co-infection with *T. vaginalis* and *C. trachomatis* increases the risk for PPROM [124,125,126]. The additive inflammatory response may be a possible explanation for the increased risk of PROM in multiple infections [126,127].

### 3.7. Actinomyces

*Actinomyces* is an opportunistic pathogen that is part of the normal vaginal flora and can be found in the oral cavity, the uterus, the lungs, and the gastrointestinal tract. It can result in infection after a break in the normal defenses of the mucosa [128]. Actinomycosis is found in pregnancy very rarely, but according to a recent review, if it occurs, it is mainly associated with preterm deliveries [129].

### 3.8. Candida Species

About 40% of pregnant women have vaginal colonization with *Candida* spp. This occurs because of the increased concentration of circulating estrogens and the vaginal accumulation of glycogen and other substrates [130]. *Candida species* rarely result in chorioamnionitis despite the high prevalence of vulvovaginal candidiasis in pregnancy. There is some evidence that eliminating *Candida* during pregnancy can reduce the risk of premature birth and late miscarriage, despite the fact that vaginal colonization with *Candida* is not usually linked to an increased probability of preterm delivery [130].

Chorioamnionitis caused by *Candida* spp. is very rare, less than 0.8%, and few cases have been reported [131,132]. The predominant species is *Candida albicans* (71.3% of all cases), and preterm labor as well as PROM in early preterm pregnancies (<28 weeks) are the most common clinical manifestations. Most reported cases of *Candida* chorioamnionitis have been associated with iatrogenic origins, such as cerclage of amniocentesis and IVF, while the tendency of *Candida* to form a biofilm amplifies these correlations [132,133,134]. Therefore, candidal chorioamnionitis should be considered when a suspected intra-amniotic infection develops after these interventions.

Although many case reports of preterm births due to chorioamnionitis caused by *Candida albicans* have been reported, only a few studies of small groups are available in the literature due to the infrequency of the infections [135,136]. Maki et al. reviewed the medical records of women with candidal chorioamnionitis and found that the most prevalent predisposing condition was PPROM (25.2%), while pregnancy with a retained intrauterine contraceptive device was in second place (21.1%), followed by a pregnancy after in vitro fertilization (20.3%) [137].

### 3.9. Campylobacter, Salmonella, and Yersinia

*Campylobacter*, *Salmonella*, and *Yersinia* are well-known causative agents of bacterial gastroenteritis in humans. However, the effects of infection with these bacteria during pregnancy remain largely unknown. Several types of *Campylobacter* have been linked to premature births and septic abortions [138,139,140]. Impaired embryo im-plantation, poor fetal development, and fetus resorption were observed in mice that were injected with Campylobacter at several stages of pregnancy [141]. Animal studies also show that *Yersinia* enterocolitica can trigger miscarriages [142]. During the first trimester of pregnancy, Kantso et al. evaluated the serological markers for *Campylobacter*, *Salmonella*, and *Yersinia* in the serum of 192 women who had contact with domestic animals. Researchers identified an association between preterm birth and high levels of *Salmonella* antibodies [143].

### 3.10. Sneathia

**Figure 2:**
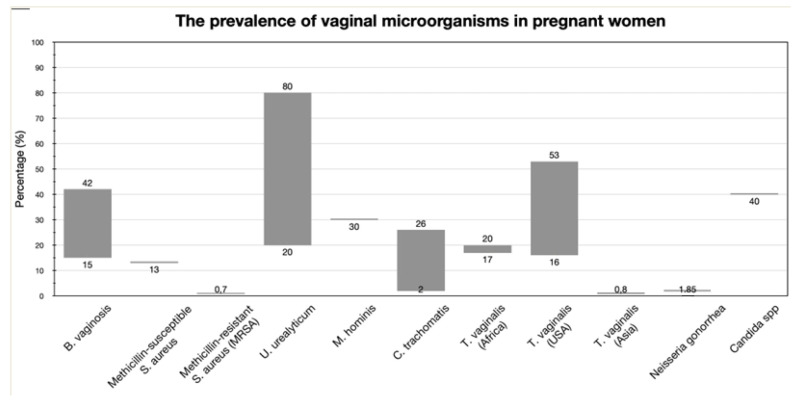
The prevalence of specific vaginal microorganisms in pregnant women. *(B. vaginosis*, mainly concerning *Gardnerella vaginalis*, varies from 15 to 42% in the general population of pregnant women, depending on the continent. Similar discrepancies are observed in colonization rates of *U. urealyticum*, *C. trachomatis*, and *T. vaginalis*, depending on the population under study.).

*Sneathia* spp. may be another pathogen related to undesirable neonatal outcomes, according to a recent review by Theis et al. Women with bacterial vaginosis and other vaginal infections are more susceptible to *Sneathia* spp. in their vaginal microbiome, a pathogen that has been linked to premature birth, chorioamnionitis, and intra-amniotic infection in research (Figure 2). *Sneathia* spp. may cause premature delivery by inflaming the cervical and vaginal epithelium and allowing pathogenic bacteria to enter. The analysis suggests incorporating *Sneathia* spp. in future vaginal microbiota and preterm birth studies, as its occurrence in diverse groups and efficient prevention and treatment options requires further study [144].

## 4. Conclusions

Genital infections during pregnancy may lead to abnormal inflammatory reactions and adverse pregnancy outcomes. In addition, there is an increased risk of adverse maternal outcome, prematurity, and neonatal morbidity and mortality. The fundamental goal of any treatment/prevention intervention should be pregnancy prolongation, the improvement of maternal–fetal health, and in cases where preterm birth is unavoidable, the amelioration of possible neonatal jeopardies. 

While placental microbiota research is ongoing, ascending vaginal bacteria are responsible for most preterm birth infections. Thus, preventing infectious preterm birth requires identifying the risk factors and developing effective treatments. Targeted microbiome-based therapies for preterm birth prevention should also be investigated. Probiotics or other microbiome-modifying agents can establish a healthy vaginal microbiota and avoid pathogenic bacteria overgrowth. Rapid and accurate vaginal infection diagnostic tests could reduce the risk of preterm birth through early detection and treatment.

Large studies are necessary for the illumination of the vaginal microbiota function and the maternal and fetal immune response in both normal pregnancies and in cases of spontaneous preterm labor. This may involve studying immune system–vaginal microbiota interactions and how dysregulation may affect prenatal outcomes.

In conclusion, future research should identify risk factors, create effective therapies, and understand how vaginal microbiome dysbiosis causes unfavorable perinatal outcomes, including premature birth. An extensive comprehension of microbial ecology and the genetic factors that regulate the reaction to infection and the inflammatory response is essential in light of the evidence that gene–environment relations may lead to preterm labor.
